# Evaluating dosimetric parameters with a plastic scintillator for megavoltage photon beam quality assurance

**DOI:** 10.1002/acm2.70276

**Published:** 2025-10-10

**Authors:** Gregory Penoncello, Bernard L. Jones, David A. P. Dunkerley, Adam Mahl, Moyed Miften, Cem Altunbas, C‐K Chris Wang, Daniel G. Robertson

**Affiliations:** ^1^ Department of Radiation Oncology University of Colorado Aurora Aurora Colorado USA; ^2^ Nuclear/Radiological Engineering and Medical Physics Programs Woodruff School of Mechanical Engineering Georgia Institute of Technology Atlanta Georgia USA; ^3^ Department of Radiation Oncology Mayo Clinic Phoenix Arizona USA

**Keywords:** monthly QA, radiation detector, scintillator

## Abstract

**Introduction:**

Linear accelerators require a large amount of data to be collected daily, monthly and annually to verify safe deliveries for patients. Different detectors have been utilized to improve the simplicity, efficiency and accuracy of the various experimental setups required to collect the necessary data resulting in reduced data collection and evaluation time. Plastic scintillators are stable and energy independent radiation detectors with high spatial resolution that emit light proportional to the amount of radiation incident on them. With an appropriate photodetector and technique to measure and analyze the light that is emitted, scintillators can be utilized to measure dosimetric parameters necessary for various monthly quality assurance requirements.

**Methods:**

A uniform cylindrical plastic scintillator imaged by a complementary metal oxide semiconductor (CMOS) camera is designed to act as a radiation detector to measure 2D projections of linear accelerator beams. Beams were delivered to the detector to evaluate machine quality assurance (QA) parameters, including beam energy, output, profile consistency and reproducibility as described by TG‐198. These measurements were compared to calculations from the Eclipse treatment planning system (TPS). Typical monthly quality assurance (QA) beams were delivered and 2D projections of the scintillation light were measured to validate the accuracy and reproducibility of this detector system for monthly QA.

**Results:**

The plastic scintillator was able to accurately characterize the radiation beam. Energy and profile measurements were reproducible and within 2%/2mm of calculations in the TPS. Output measurements had maximum variations of up to 1.3% and average differences of 0.5%.

**Conclusion:**

A simple cylindrical plastic scintillator and CMOS camera radiation detector setup was designed and tested for measuring monthly QA dosimetric parameters specified by TG‐198 with accurate and reproducible output, energy and profiles measurements. This method reduces the number of measurements required, allowing multiple parameters to be evaluated in a single beam delivery.

## 1 INTRODUCTION

External beam radiation therapy utilizing a conventional C‐arm linear accelerator (LINAC) requires periodic evaluation of mechanical and dosimetric parameters to ensure a safe and accurate treatment. These include different types of checks, or quality assurance (QA), that must be completed with different frequencies, including on a daily, monthly and annual basis. The American Association of Physicists in Medicine (AAPM) Task Group's (TG) 40, 142 and 198 as well as Medical Physics Practice Guideline (MPPG) 8.b describe in great detail the numerous tests and methods that are recommended to verify the consistency and accuracy of a LINAC.^1^, ^2^, ^3^, ^4^ For monthly QA the dosimetric parameters that are generally evaluated include measuring and tracking the output of the different treatment beams. This includes verifying that the output remains constant for multiple dose rates as well as in a beam hold scenario such as with gating. The energy of the beams and the beams’ profiles need to be evaluated and remain consistent.^1^, ^2^, ^3^, ^4^ These measurements are compared to baseline values to ensure the stability of these dosimetric attributes of the LINAC.^2^, ^3^, ^4^

Numerous devices are capable of taking these measurements. The most common technique to verify output is to use a farmer‐type ionization chamber in solid water and take a point measurement at a depth that correlates with the calibration geometry as described in TG‐198. To measure energy, a single point measurement at a separate depth is commonly taken to create a ratio with the output measurement point. To measure the constancy of beam profiles, commonly a two dimensional (2D) array of either micro ionization chambers or diodes are used. These measurements are taken at specified depths by using different thicknesses of solid water to create buildup. Some institutions have been able to take measurements more efficiently using the IC Profile (Sun Nuclear Melbourne, FL) to measure output and profiles at the same time.^5^, ^6^ Energy can be measured using specialized devices that incorporate a gradient of buildup material into the beam called quad wedge plates (Sun Nuclear Melbourne, FL) with the IC Profiler in a separate measurement.

Scintillating materials have been used as radiation detectors for many years.^7^, ^8^, ^9^ The basic mechanism for a scintillator‐based radiation detector is converting high energy from an incident quanta of radiation, photons or other particles into lower energy photons in the visible spectrum. These lower energy photons are more easily measured and quantified with common photo‐detectors. When the high energy radiation interacts in the scintillator material, electrons in the material become excited into higher energy states. When the electrons relax back into the ground state, photons are emitted through a process called fluorescence. In general, the amount of light that is converted and ultimately emitted is proportional to the amount of energy that the scintillator absorbs.

There are a few important properties for a given scintillator to be useful. They should have a high quantum efficiency, meaning that the material yields a large amount of visible or near visible light relative to the energy that it absorbs. It should have a fast emission time, meaning that it emits the light very quickly after absorbing the radiation. Finally it should minimize re‐absorption any of its own emitted light photons, that is, have a low Stokes shift.^7^, ^8^ Plastic scintillators are a specific type of organic scintillating material, which can be manufactured with all of these desired properties and are additionally useful because they are durable, easily constructed to any desired shape and size and are tissue equivalent. Additionally, if coupled with an appropriate resolution photodetector they can have a very high resolution and provide very precise 2D dose projection maps.

The use of plastic scintillators in radiation therapy has been extensive. Three dimensional scintillator detectors have been primarily used in machine QA for proton beams to measure the scanning spot position and range.^10^, ^11^ Scintillator detectors have been used for patient QA with volumetric scintillators for proton beams for prostate plans.^12^ For photon radiation measurements, a scintillator and plenoptic camera were used to measure 3D IMRT photon fields.^13^ A 1mm plastic scintillator for single target VMAT SRS plans.^14^ Log files and plastic scintillator measurements were used to reconstruct dose for an MRI‐LINAC.^15^ An array of plastic scintillators with optical fibers were used to measure small field dosimetric parameters.^16^, ^17^ 29 plastic scintillators with optical fibers were used to measure beam dosimetric parameters.^18^ A plastic scintillator was used to compare static beams for 10 × 10 fields and other static MLC configurations.^19^ This scintillator camera setup also measured 2D IMRT and VMAT dose distributions with an array of plastic scintillator detectors and began moving towards 3D measurements.^20^

In this report a newly designed plastic scintillator detector has been constructed, tested and evaluated as a reproducible, consistent and simple method to obtain all of the dosimetric measurements required in a standard medical physics monthly QA program. A cylindrical, monolithic plastic scintillator was housed in a light tight box with a complementary metal oxide semiconductor (CMOS) camera to measure the emitted light. The measured light in the camera is a 2D projection of a 3D dose distribution and compared to calculations or other baseline measurements for dosimetric verifications.

## 2 MATERIALS AND METHODS

### 2.1 Detector construction

**FIGURE 1:**
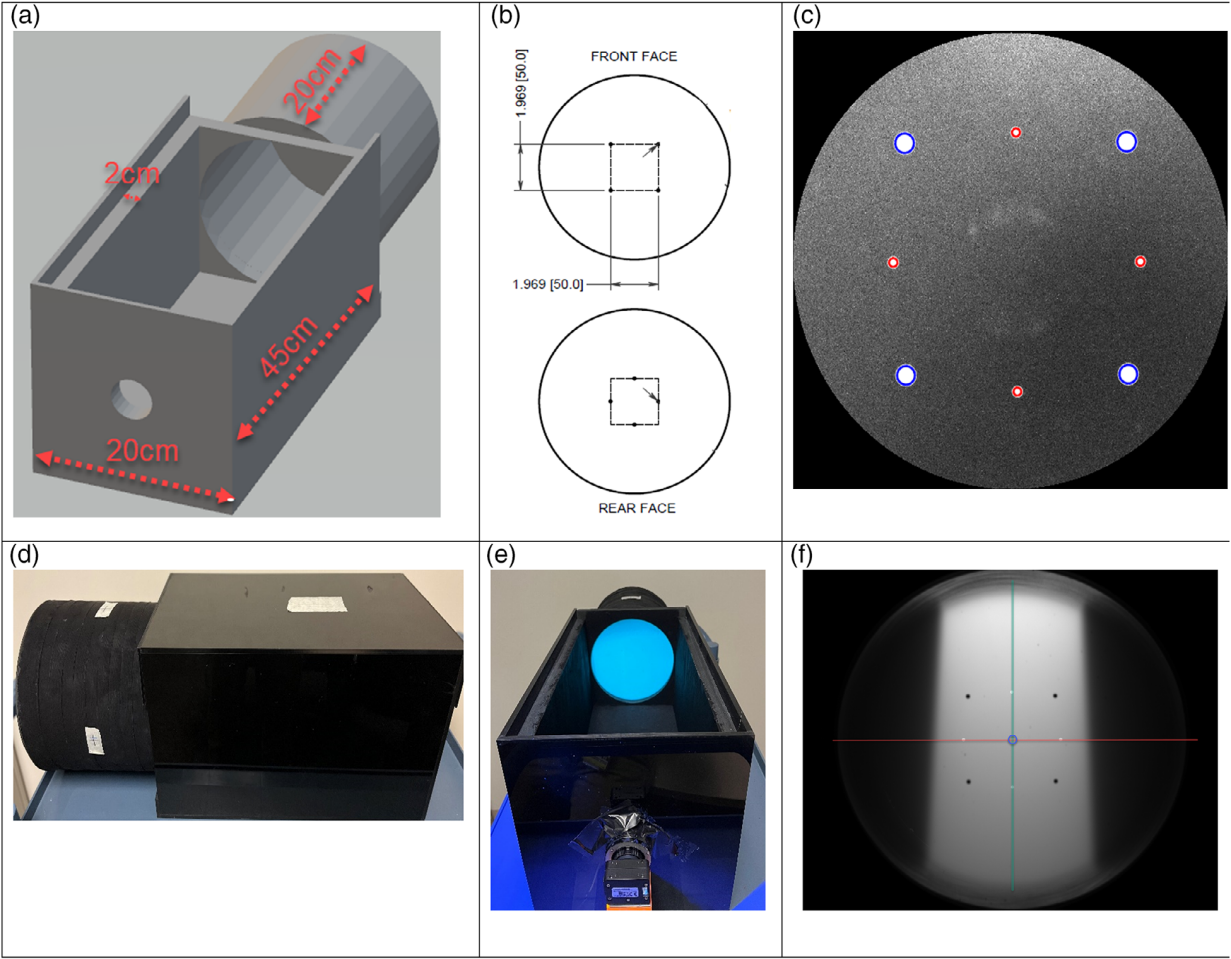
Images of different aspects and designs of the detector. (a) The geometric design of the detector. (b) The positioning of the score marks in mm on the front and rear face of the cylinder. (c) The localization of the score marks in the ambient open light field image to determine the center of the phantom relative to the camera. (d) The completed enclosed detector. (e) The scintillator cylinder in the open box with a UV flashlight shining on it from the direction of the camera. (f) A composite image of a 10 × 10 cm 6× field of 10 MUs collected with the camera. The red line indicates the profile, the green line indicates the PDD and the blue circle indicates the point at which the PDD and profiles were normalized. The blue circle also indicates the location where the calibration curve was created, as well as the location where the output was measured.

The plastic scintillator detector was enclosed in a light tight box and Styrofoam casing with the CMOS camera positioned 45 cm away from the front circular face of the cylindrical scintillator as shown in Figure 1a. A plastic scintillator was manufactured into a right equilateral cylinder of 20 cm diameter and 20 cm length made of commercially available scintillating material EJ‐260 (Eljen Technologies, Sweetwater TX). This material was chosen due to its fast rise and decay time of 1.5ns and 9.2ns, its scintillation efficiency of 9200 photons/1 MeV e^−^ and its light attenuation length of 350 cm. These properties ensured a high signal‐to‐noise ratio to be measured with the CMOS camera that is used for light collection.

All of the cylinder's surfaces except for the flat surface facing the camera were painted with black acrylic paint to reduce internal reflection. A layer of Styrofoam 2 cm thick was added to the outer surface of the scintillator to further enhance light tightness of the system while also providing protection and support for the plastic scintillator itself. The light tight box was also padded with Styrofoam, all of which was then painted with several coats of non‐reflective black acrylic paint. This design allowed only photons that were emitted directly in the direction of the camera to be counted.

Four score marks painted white were oriented on both the front and back circular faces of the scintillator each in the shape of a 5 cm × 5 cm square, oriented 45° in relation to each other as depicted in Figure 1b. These score marks were used for localization and finding the center of the phantom relative to the camera as depicted in Figure 1c. The score marks were also useful in ensuring the focus of the camera was at the appropriate depth of 10 cm into the cylinder. The CMOS camera, a Chameleon3 (Teledyne FLIR, Wilsonville OR) was mounted to an HFSA16 objective lens (Fujinon, Saitama City Japan) with a focal length of 16 mm and f stop of 1.4 so that the field of view of the camera fully encompassed the front of the 20 cm diameter scintillator. Figure 1d,e show pictures of the completed detector with the scintillator and camera positioned on the box detector.

### 2.2 Measurements

Attix describes important characteristics to quantify dosimeters, including precision and accuracy, dose sensitivity, stability and energy dependence.^21^ Various measurements were taken to quantify these features. The assembled box was positioned such that the center of the scintillator was at isocenter, creating a 100 cm source to axis distance (SAD) setup with the geometric centroid of the detectors active volume. This alignment was done using the lasers and crosshairs on the detector, consistent with standard monthly QA setup practices. The camera is positioned in line with the gantry head and scintillator. Various field sizes, energies and MUs were shot at different times with different setups to measure dosimetric parameters such as beam energy, output, profile consistency and reproducibility as depicted in Table 1. These were taken with the gantry positioned upright, or gantry zero. Figure 1f shows an example of a resulting composite image from the detector following irradiation of a 6MV beam for a 10 × 10 cm field size.

**TABLE 1:** Measurements taken for comparison for Profiles and PDDs, to build the calibration curve, for output consistency at all other energies and to compare reproducibility. PDDs were only taken for square fields.

Profiles and PDDs		
Energy	Field sizes	MU
6×	1 × 1^†^, 5 × 5, 5 × 1, 1 × 5, 10 × 10^†^, 10 × 1, 1 × 10, 15 × 15^*^, 15 × 1, 1 × 15, 30 × 30, 30 × 1, 1 × 30	10
6 × FFF	1 × 1^†^, 5 × 5, 5 × 1, 1 × 5, 10 × 10^*^, 10 × 1, 1 × 10, 15 × 15, 15 × 1, 1 × 15, 30 × 30, 30 × 1^†^, 1 × 30^†^	10
10×	1 × 1, 5 × 5^†^, 5 × 1^†^, 1 × 5^†^, 10 × 10, 10 × 1, 1 × 10, 15 × 15, 15 × 1, 1 × 15, 30 × 30^*^, 30 × 1, 1 × 30	10
10 × FFF	1 × 1^*^, 5 × 5, 5 × 1, 1 × 5, 10 × 10, 10 × 1, 1 × 10, 15 × 15^†^, 15 × 1^†^, 1 × 15^†^, 30 × 30, 30 × 1, 1 × 30	10
15×	1 × 1, 5 × 5^*^, 5 × 1, 1 × 5, 10 × 10, 10 × 1^†^, 1 × 10^†^, 15 × 15, 15 × 1, 1 × 15, 30 × 30^†^, 30 × 1, 1 × 30	10
Calibration curve		
Energy	Field Size	MU
6×	10 × 10	10
6×	10 × 10	25
6×	10 × 10	50
6×	10 × 10	100
Output consistency		
6×	10 × 10	100
6×	10 × 10	100
6×	10 × 10	50
6×	10 × 10	25
6×	10 × 10	10
10×	10 × 10	10
10×	10 × 10	25
6 × FFF	10 × 10	100
6 × FFF	10 × 10	50
10 × FFF	10 × 10	25
15×	10 × 10	25
Reproducibility		
6×	10 × 10	100
6×	10 × 10	100
6×	10 × 10	100
6×	10 × 10	10

* PDD for field size displayed.

^†^ Profile for field size displayed.

Prior to collecting the radiation measurements, a background dark image and an open ambient light image with the top of the detector off for positional verification of the scintillator relative to the camera were taken. Figure 1c shows an example of the ambient open light image used for localization. Measurements were taken for each energy of 6 MV (6×), 6 MV flattening filter free (6 × FFF), 10 MV (10×), 10MVFFF (10 × FFF) and 15 MV (15×). Field sizes ranged from 1 × 1 to 30 × 30 square fields and rectangle fields of similar dimensions. Table 1 indicates the field sizes measured for each respective beam. Each of these beams were delivered with 10 monitor units (MU). A single measurement for each field size could be used to compare both the profile and PDD since a full 2D projection was measured each time. Additionally inline profile measurements were taken for each corresponding crossline measurement with the couch rotated to 90°. The rectangular fields are measured to verify the quality of the detector by minimizing the error in refraction described below in Section 2.3. Square fields are measured to be validated for monthly QA utilization.

To correlate measured light to dose, a calibration curve is needed. To produce this calibration curve a 6×, 10 × 10 beam was delivered with different MUs. Table 1 shows the measurements taken to create the calibration curve. The dose measured at the center of the phantom as shown in Figure 1f was used to make a calibration curve based on values calculated in Eclipse (v15.6 Varian Medical Systems, Palo Alto CA) using the AcurosXB_15605 dose algorithm. A subset of similar measurements was then taken for all other energies to evaluate dose accuracy using the single 6× calibration curve. Table 1 shows the measurements taken for dose accuracy.

To ensure measurement reproducibility, three separate measurements of 6×, 10 × 10 field of 100 MUs and one of 10MU were collected and the PDD and crossline profile were plotted. To ensure the sensitivity of the detector for symmetry, a 6 × 10 × 10 field of 10 MU was delivered with a 2° gantry rotation and compared to a measurement with no rotation. Similarly, to ensure sensitivity of beam quality, an addition comparison of PDDs of the device placed at SAD and a measurement with the detector 10 cm further away from the source. An explicit comparison of a beam delivering 49 MU and 51 MU compared to 50 MU was also completed to ensure the sensitivity of the detector with small changes in output.

The camera settings were constant for all image acquisitions. The acquisition mode was continuous at a 35.66 Hz frame rate. No exposure compensation was used and a gain of 15 dB was applied. Finally, the exposure mode was timed at 20 ms with measurements manually turned on and off before and after the beam was delivered respectively. The spatial resolution of the camera at the central axis was 0.11 × 0.11 mm.^2^ The refraction corrections adjust the resolution off axis as a function of distance from the central axis as described below.

### 2.3 Image processing

The CMOS camera has multiple imaging artifacts must be accounted for in order to accurately map the light collected in reference to the experimental setup. These parameters, as described by Robertson et al.,^22^ are correcting for background, salt and pepper noise, image distortion, vignetting and refraction. To correct for background an image was taken before every session with the ambient light setting for each experimental setup and was subtracted appropriately. To correct for salt and pepper noise, an edge preserving bilateral filter to a 3 × 3 region around each pixel is used on the collected image. This filter uses a weighted average of intensities of the surrounding pixels to adjust each pixel to smooth the image and reduce noise.

To correct for vignetting, Equation 1 was used^22^, ^23^

(1) $$\;V_{i,j}=cos^{4}\;\left(\theta_{i,j}\right)=\frac{a^{4}}{{\left(a^{2}+d_{i,j}^{2}\right)}^{2}}$$

Here, a is the distance from the exit pupil of the camera to the principal point or center pixel of the camera image. This must be experimentally measured. *d* is the distance from the center pixel to pixel (*i*,*j*). *θ* is the angle between the optical axis (along distance a) and the trajectory of a photon striking pixel (*i*,*j*). In this design, the vignetting was measured using a uniform light source to see the decrease in intensity as a function from the center of the camera. The measured function was fit with Equation (1) to obtain the appropriate value for a. Equation (1) then provides a multiplicative correction to the intensity of light collected for each pixel as a function of pixel position relative to the center pixel.

To correct for lens distortion, a 14 × 10 square black and white checkerboard was imaged at varying positions to cover the field of view of the CMOS camera. These images were used in the Matlab Camera Calibration Toolbox version 2024a (MathWorks, Natick MA) to provide the intrinsic and extrinsic parameters of the camera. These parameters were applied on a frame‐by‐frame basis to correct for distortions in the image.^24^, ^25^

To correct the positional axis associated with position of light collected due to refraction, a pin hole camera technique was used, as described by Robertson et al.^22^ This correction was applied radially from the central axis of the camera. The coordinate system for each pixel was adjusted relative to the center of the cylinder. Equation (2) below shows how this is calculated for all pixels.

(2) $$\;d_{f}=d_{s}\;*\tan\left\{a\mathrm{rcsin}\left[\left(\frac{n_{1}}{n_{2}}\right)*\sin\left[\arctan\left(\frac{r}{d_{c}}\right)\right]\right]\right\}$$

where *d_f_* is the refraction shift in the axis of the beam, *d_s_* is the depth in the phantom and defined below in Equation (3), *n*_1_ is the refractive index of air, *n*_2_ is the refractive index of the scintillator, *r* is the radius from the center of the camera and *d_c_* is the distance from the camera to the phantom.

**FIGURE 2:**
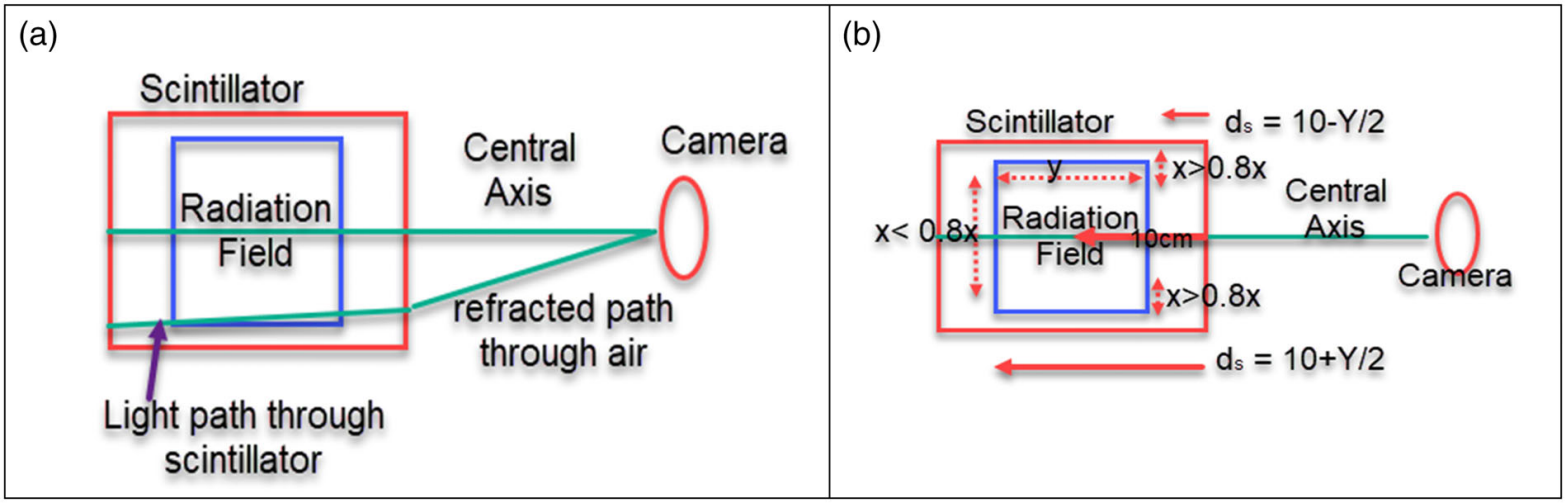
Diagrams of refraction of light through the plastic scintillator. (a) Top‐down diagram of an incident radiation field, which produces light traveling through the plastic scintillator collected by the camera. A pixel will collect light from the penumbra of the beam furthest from the camera and full intensity of the beam closer to the camera. (b) How d_s_ is calculated relative to the geometry of the radiation beam incident on the plastic scintillator.

The complications of refraction correction come from the fact that the incident radiation beam on the scintillator has a width in the direction of the camera. The image collected is a 2D projection over the entire width. Thus, the light accumulated in each pixel comes from a collection of different depths. Figure 2a shows how different aspects of the beam sum up in a given pixel. In general, only a single depth can be corrected for refraction. Equation (3) as d_s_ in Equation (2) allows for two depths to be corrected for refraction. This technique also ensures the largest refraction is corrected for and that the light on the periphery of the beam, which is mostly measured in the front of the detector is not overcorrected.

The beams in this study were all centered on the middle of the scintillator, so refraction correction for the center of the beam perpendicular to the camera was made at the position furthest from the camera. For the penumbra of the beam this correction was made at the position of the beam that is closest to the camera. This split correction method results in the inner 80% of the nominal field being corrected to the position of the field furthest from the camera and the remaining 20% of the nominal field size is corrected to position of the beam closest to the camera. Equation (3) shows the equations.

(3) $$\;d_{s}=\left\{\begin{array}{c} 10+\frac{Y}{2}\;for\;x<0.8X\\ 10-\frac{Y}{2}\;for\;x>0.8X \end{array}\right.\;$$

where *Y* is the nominal field size along the depth axis of the camera and *X* is the nominal field size orthogonal to the camera scintillator axis. Figure 2b shows a diagram of the depth used for refraction correction.

Each of these corrections was applied to each frame of every measured beam. Each frame was then summed together for a total composite image of light collected in a 2D projection.

### 2.4 Calculations and comparisons

Experimental measurements were compared to expected TPS values (as described in Section 2.2). A Computed Tomography (CT) simulation scan of the detector was taken with 2 mm slice thickness. This scan was used to create a treatment plan with each beam delivered to the scintillator detector modeled and calculated with a 2 mm dose calculation grid size. The calculated 3D dose maps were exported as a Digital Imaging and Communications in Medicine (DICOM) file and read into MATLAB R2024a (MathWorks, Natick MA). The 3D dose data was collapsed in a parallel trajectory and summed to create a 2D projection in the direction of the camera. This projection was then compared to experimental measurements for PDDs and profiles by using the implanted score marks in the scintillator for registration purposes.

PDD measurements were compared down the center of the scintillator and normalized to the central pixel of the scintillator as shown by the green line in Figure 1f. Profile measurements were also compared along the central axis of the scintillator and normalized at the center pixel in the scintillator as visualized by the red line in Figure 1f. Light reflection was enhanced by the white dash marks. This was subtracted out and linear interpolated between points on either side. Output measurements were compared at the center of the scintillator and correlated to the calibration curve that was created as detailed in Section 2.2. The measured output was then corrected with daily output measurements using the Daily QA3 (DQA3) device from Sun Nuclear (Melbourne FL) on the same day of measurement to minimize the effects of daily linac output fluctuations.

## 3 RESULTS

All measurements were collected and evaluated with the results showing agreement within 2%/2mm 1D gamma calculations of the TPS for profiles and PDDs. The output measurements were consistent and reproducible within 1.5%. For smaller field sizes, the collected images were generally more noisy due to less light being detected within each frame. It was noted that the white score marks in the rear face caused an increase in signal as the light reflected off them, so this increase was subtracted out.

### 3.1 Percent depth dose

**FIGURE 3:**
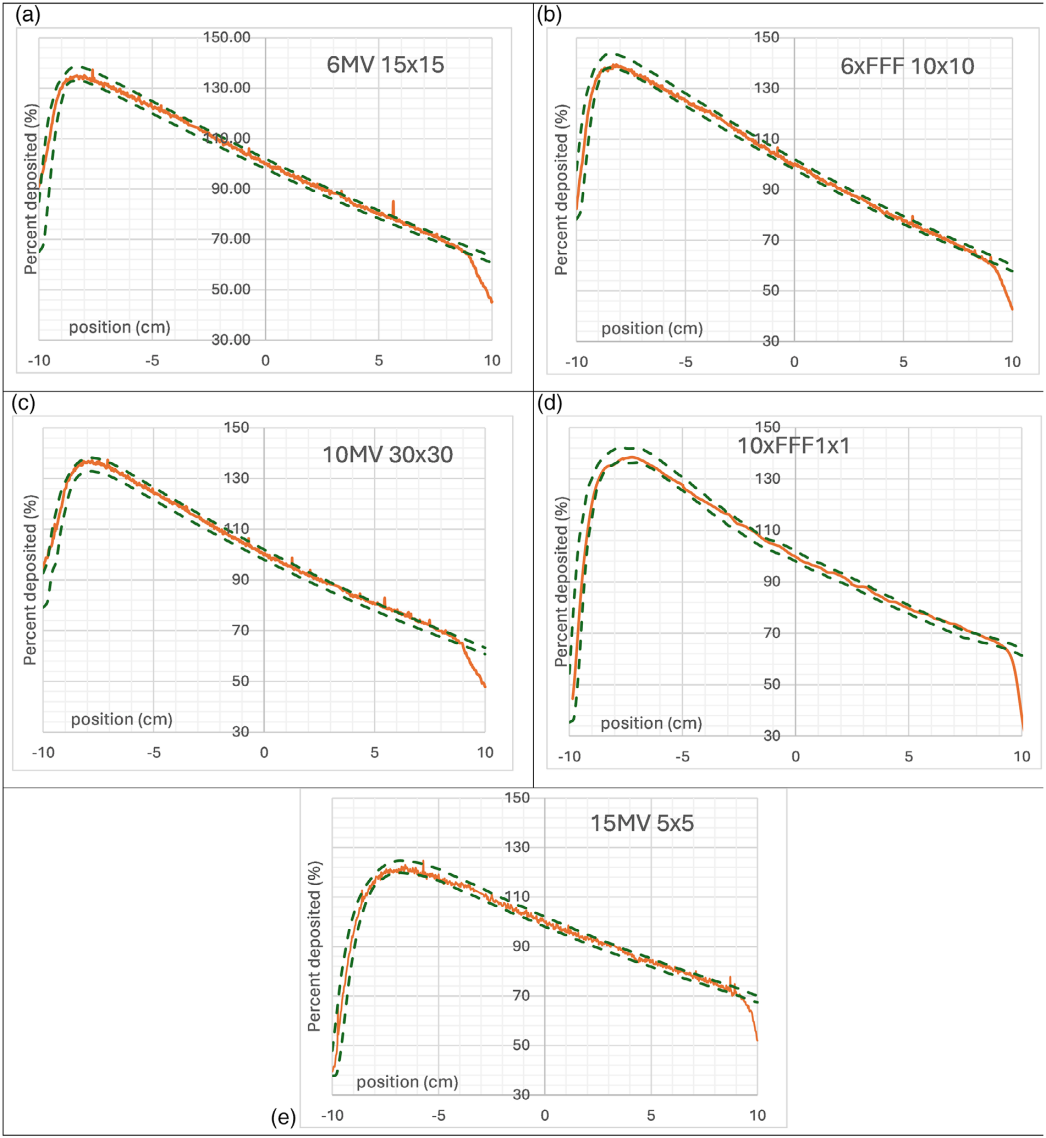
PDD measurements (orange) for all energies and varying field sizes with overlaid 2%/2 mm gamma lines from calculations from eclipse. All measurements and calculations are normalized to the center of the scintillator. (a) 6 × 15 × 15 cm, (b) 6 × FFF 10 × 10 cm, (c) 10 × 30 × 30 cm, (d) 10 × FFF 1 × 1 cm, (e) 15 × 5 × 5 cm.

Each of the measured PDDs correlated in general within 2%/2mm 1D gamma calculation of expected values. Figure 3 shows plots of varied results of energy and field size. The measured PDD curve is overlaid with the 2%/2mm gamma curve calculated in the TPS for comparison.

### 3.2 Crossline and inline profiles

**FIGURE 4:**
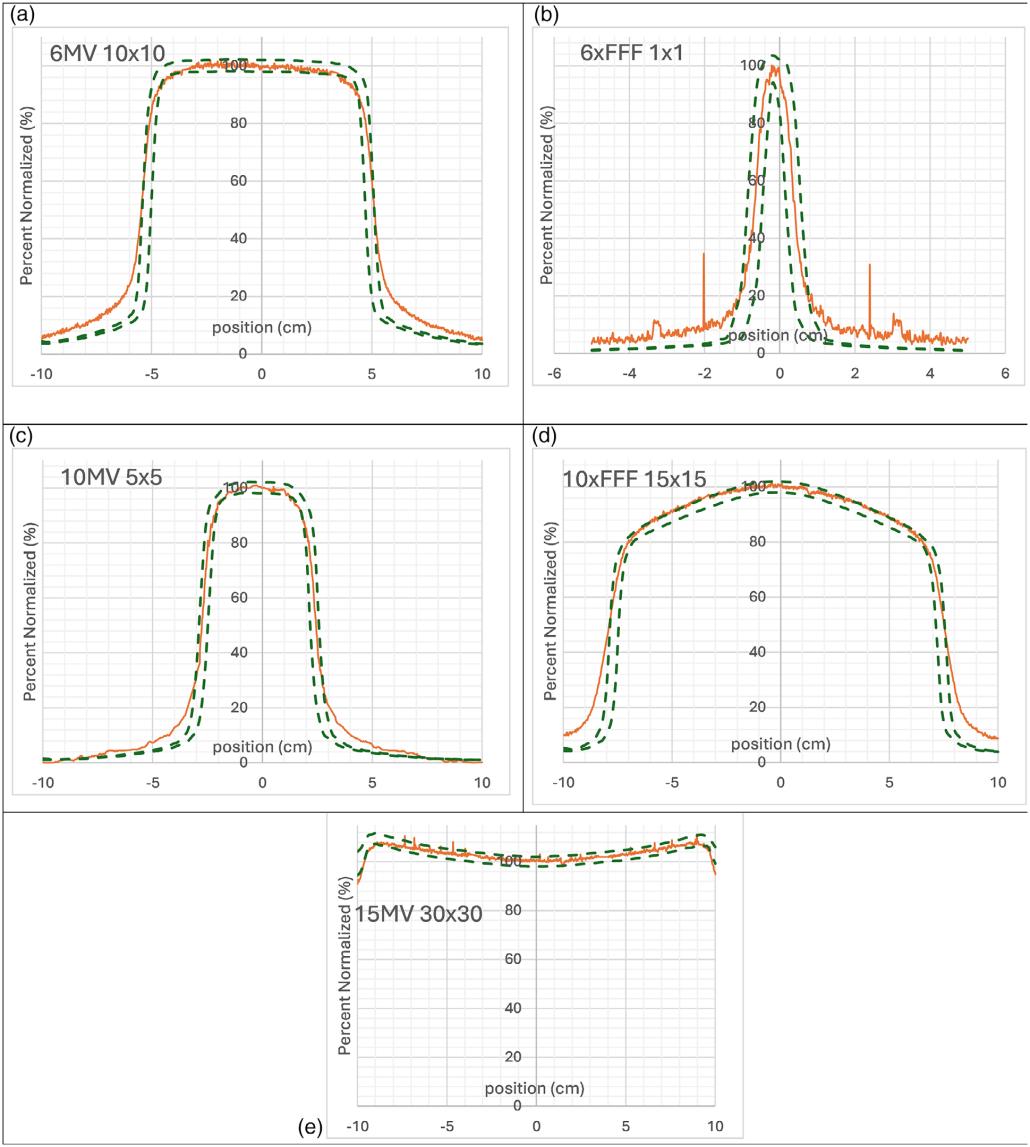
Crossline profile measurements (orange) for all energies and varying square field sizes with overlaid 2%/2 mm gamma lines from calculations from Eclipse. (a) 6 × 10 × 10 cm, (b) 6 × FFF 1 × 1 cm, (c) 10 × 5 × 5 cm, (d) 10 × FFF 15 × 15 cm, (e)15 × 30 × 30 cm.

**FIGURE 5:**
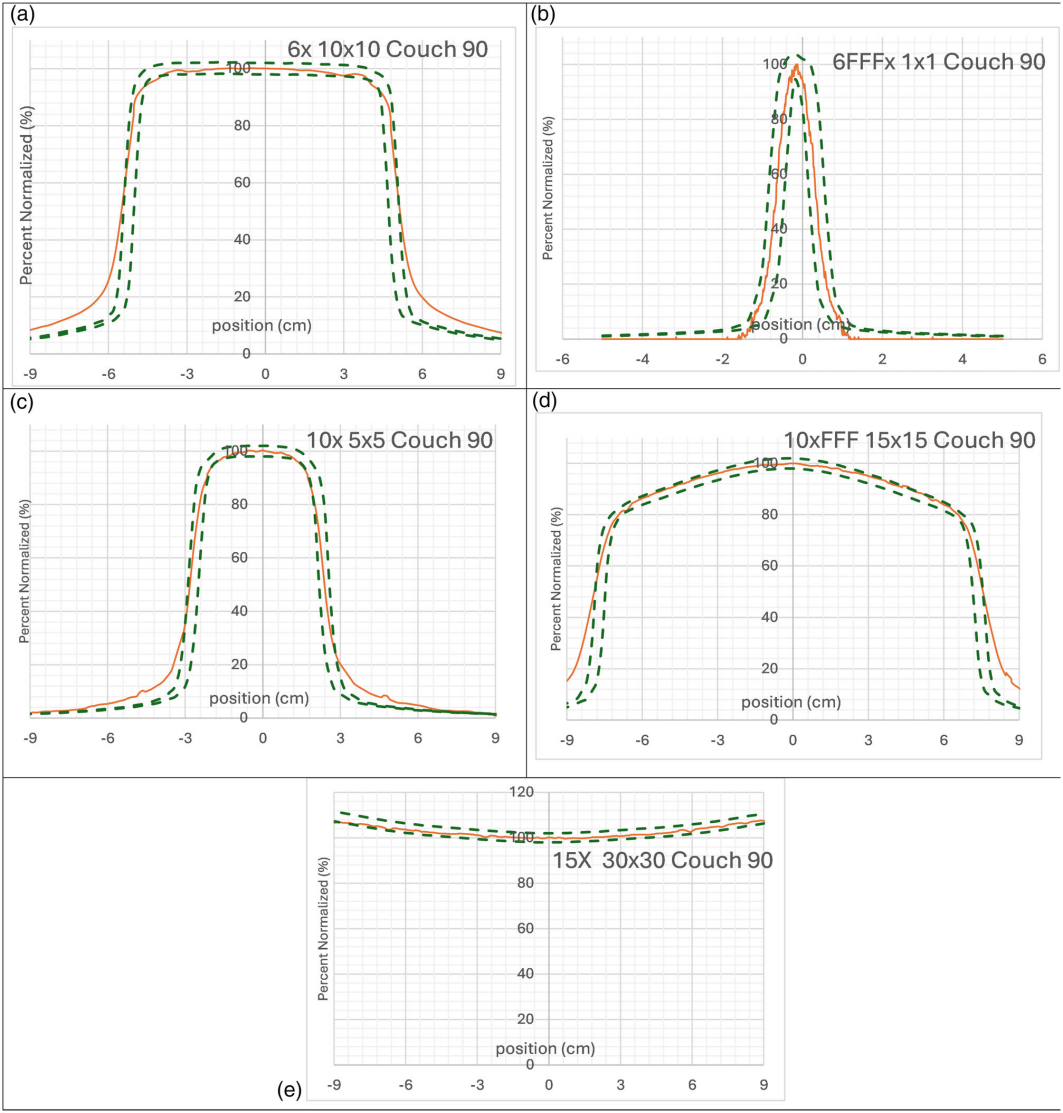
Inline profile Measurements (orange) for all energies and varying square field sizes with overlaid 2%/2 mm gamma lines from calculations from eclipse. (a) 6 × 10 × 10 cm, (b) 6 × FFF 1 × 1 cm, (c) 10 × 5 × 5 cm, (d) 10 × FFF 15 × 15 cm, (e) 15 × 30 × 30 cm.

The crossline and inline profiles of each square field delivered beam agreed within 2%/2mm gamma calculation in the open part of the field, but fell out at different points in the penumbra region. Figures 4 and 5 shows a subset of results for various field sizes and energies with the plot of each profile overlaid with the 2%/2mm curve as calculated from the TPS. There is disagreement in the penumbra region that shows dependence on the field size but not energy. Disagreement in the penumbra region with the 2%/2mm gamma calculation occur at 20% of the maximum intensity for 1 × 1 fields, 30% for 5 × 5 fields, 50% for 10 × 10 fields and for 15 × 15 fields.

**FIGURE 6:**
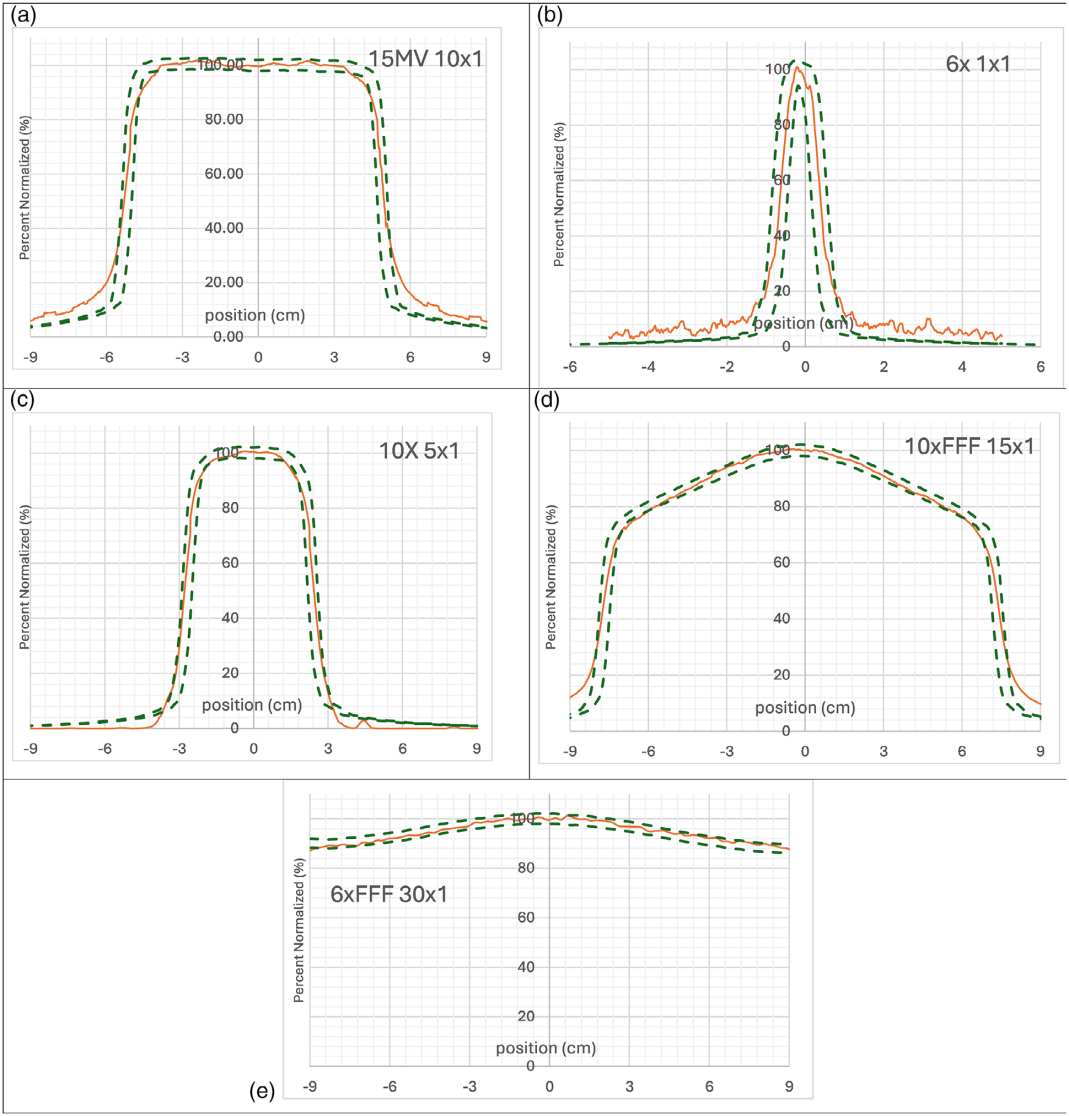
Crossline profile measurements (orange) for all energies and varying rectangle field sizes with overlaid 2%/2 mm gamma lines from calculations from eclipse. (a) 15 × 10 × 1 cm, (b) 6 × 1 × 1 cm, (c) 10 × 5 × 1 cm, (d) 10 × FFF 15 × 1 cm, (e) 6 × FFF 30 × 1 cm.

**FIGURE 7:**
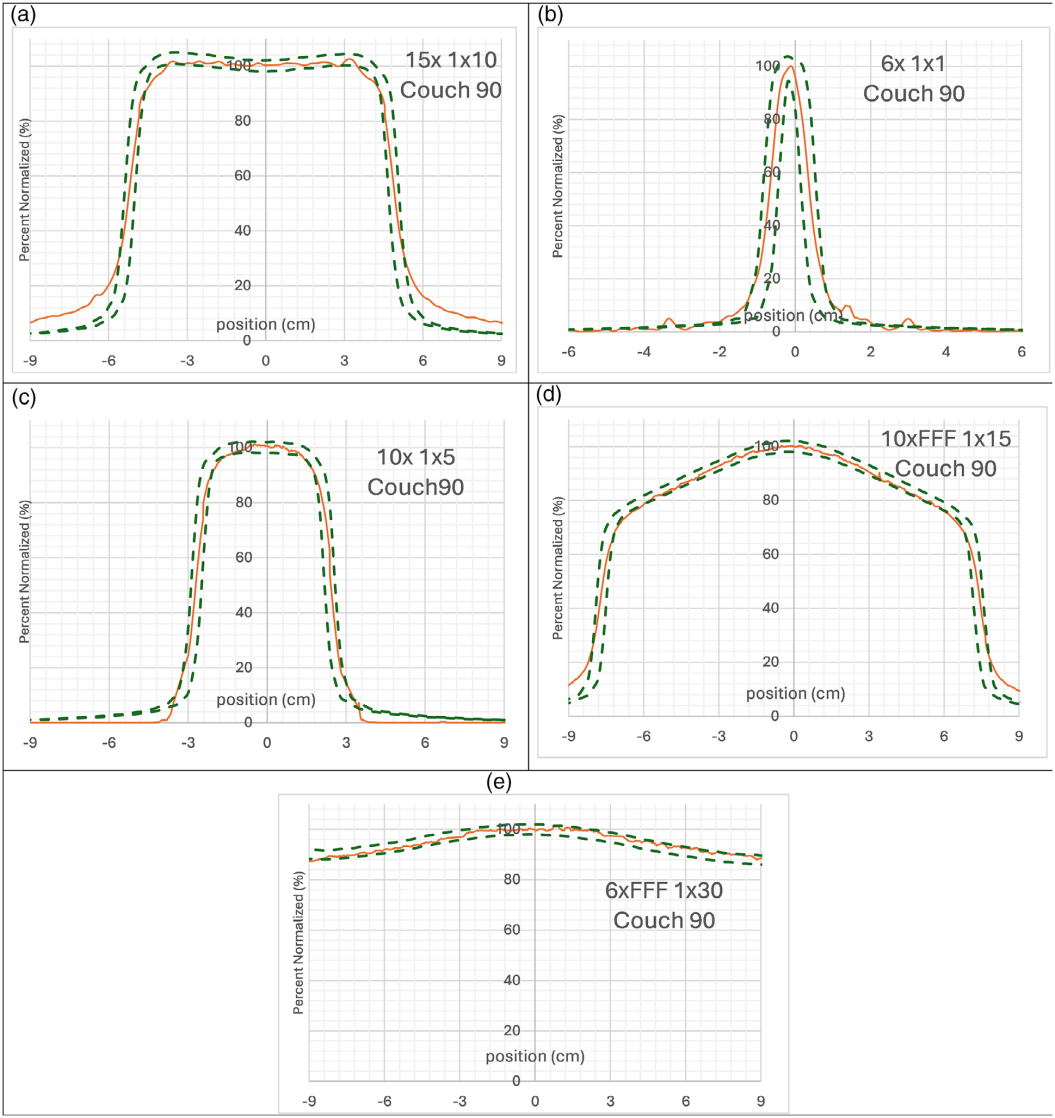
Inline profile measurements (orange) for all energies and varying rectangle field sizes with overlaid 2%/2 mm gamma lines from calculations from eclipse. (a) 15 × 1 × 10 cm, (b) 6 × 1 × 1 cm, (c) 10 × 1 × 5 cm, (d) 10 × FFF 1 × 15 cm, (e) 6 × FFF 1 × 30 cm.

The crossline and incline profile for the rectangular fields did not have issues in the penumbra region as the square fields and agreed within 2%/2mm. For some fields the very periphery of the edges had some mismatch. Figures 6 and 7 shows a subset of results for the various field sizes and energies of each crossline and inline profile with the 2%/2mm curve as calculated from the TPS.

### 3.3 Reproducibility and sensitivity

**FIGURE 8:**
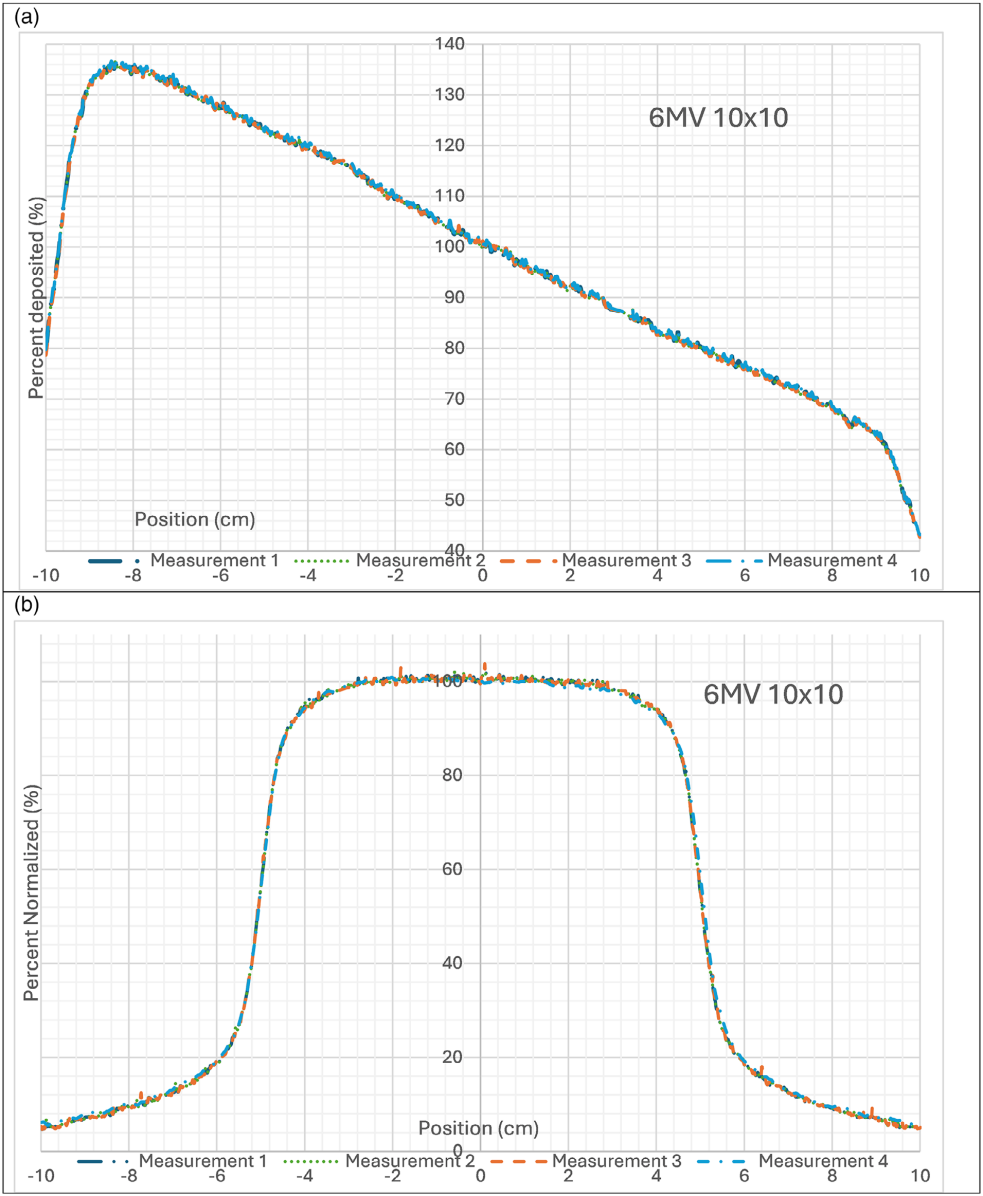
Reproducibility of four separate measurements of a 6 × 10 × 10 field. Measurements 1–3 delivered 100MU and Measurement four delivered 10MU (a) PDD measurements, (b) crossline profile measurements.

The reproducibility for each measurement was found to be consistent and acceptable. Figure 8 shows the agreement of three 100MU deliveries and one 10MU delivery of a 10 × 10 field for 6x for PDD and crossline profiles overlaid on top of each other. Each single point within the four different measurements differed from each other by a maximum of 1.5%, an average of 0.12% and a standard deviation of 0.36% for PDDs. Each single point within the four different measurements differed from each other at a maximum of 2.1%, an average of 0.05% and a standard deviation of ± 0.39%for crossline profiles.

**FIGURE 9:**
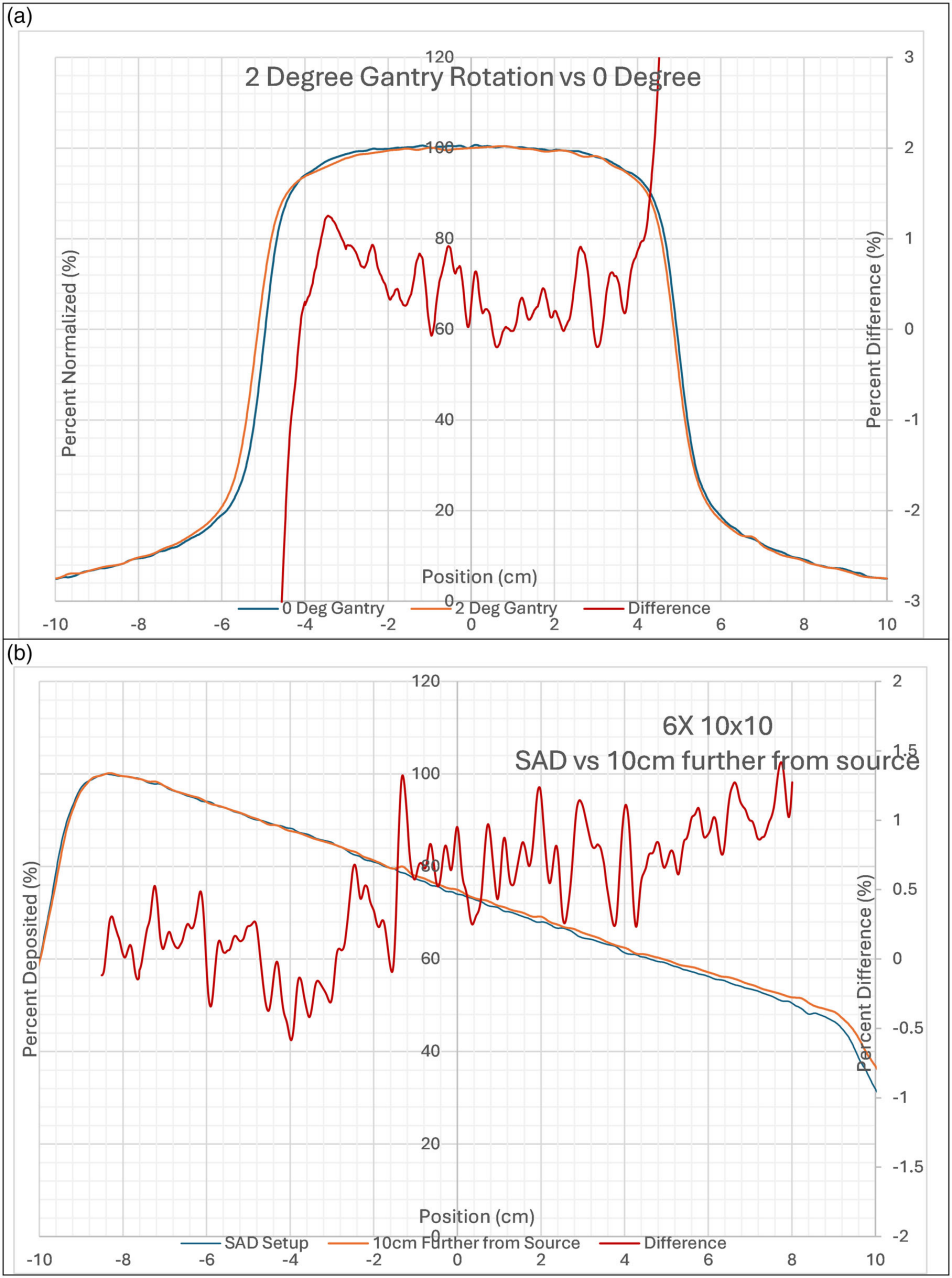
Displays the sensitivity of the detector (a) change in symmetry for 6 × 10 × 10 field due to a 2° gantry rotation Blue = 0° gantry, Orange = 2° gantry and Red = difference between the two (b) change in beam quality as a result of increased SSD of 10 cm versus SAD measurement. Blue = SAD setup, Orange = 10cm increase in SSD measurement and Red = difference between the two.

The detector showed good sensitivity for changes in symmetry, beam quality and output. With a 2° gantry rotation about a 0.6% change in symmetry was measured compared to the zero‐rotation measurement. The PPD10x changed about 0.95% with the detector extended 10cm further from the source compared to the measurement taken at 100cm SAD. These results are shown in Figure 9 and correlate with calculated results of 0.5% and 0.92% respectively. When 49MU and 51MU was delivered and compared to a 50MU delivery, it measured 2.1% lower than the 50MU measurement and 2.18% higher than the 50 MU measurement respectively. This is as expected, since 49MU is 2% lower and 51MU is 2% than 50MU.

### 3.4 Output measurements

Output measurements showed acceptable agreement between the calibration curve and the experimental light signal, given the measured relative output of the machine for that day for each energy. Scintillator measurements showed an average value of 0.55% agreement with calculations from the TPS with a 0.76% standard deviation. After taking into account the DQA3 output measurements this average agreement was 0.52% with a 0.63% standard deviation. All results were within 1.0% agreement except for 10MVFFF which showed just under 1.3% agreement with the TPS. Table 2 shows the results of varying output measurements at different MUs for 10 × 10 field sizes for different energies. It also shows the daily output measurement of the DQA3.

**TABLE 2:** The measured raw scintillator outputs compared to the TPS, the daily linac output measured with the dailyQA3 and the scintillator output corrected with the daily linac output compared to the TPS.

Energy	MU	Raw scintillator output percent difference	DailyQA3 output	Corrected scintillator output percent difference
6 MV	100	−0.071%	0.18%	−0.25%
6 MV	100	0.047%	0.18%	−0.13%
6 MV	50	−0.31%	0.18%	−0.49%
6 MV	25	0.79%	0.34%	0.45%
6 MV	10	−0.72%	−0.35%	−0.37%
10 MV	10	0.51%	−0.20%	0.71%
10 MV	25	0.17%	0.47%	−0.30%
6 × FFF	100	0.84%	0.22%	0.62%
6 × FFF	50	−0.19%	0.22%	−0.41%
10 × FFF	25	2.11%	0.85%	1.26%
15×	25	0.25%	1.00%	−0.75%
Average		0.55% ± 0.76%		=0.52% ± 0.63%

## 4 DISCUSSION

The results of the dosimetric evaluation show that this scintillator camera detector can be utilized to effectively and accurately measure dosimetric parameters needed for a monthly QA program. The accuracy of this device, when compared to the TPS generated baselines for both the beam profiles and the PDDs, are within the guidelines of TG‐142 and TG‐198 for thresholds of 2% of device baseline for output and profile consistency and ± 1% of a PDD point from baseline measurement.^1^, ^2^, ^3^, ^4^ The measurements in this report were compared to the TPS, while the TG reports note that these measurements should be compared to baselines. MPPG 8b. discusses a transition to a TPS comparison for beam tolerance as opposed to a baseline measurement.^4^ The reproducibility capability of this detector shows that it would be an acceptable device to use in either a baseline comparison or a TPS comparison and is sensitive enough to detect subtle variations in all dosimetric parameters measured. This will allow for a single shot for each energy to verify output, energy and crossline profiles. A second shot with the couch at table 90° or 270° provides the in‐plane profile measurements. Since the collected measurement is analyzed as a projection it means that there is an indirect correlation in the inline direction, it is just not explicitly quantified without the couch rotation measurement. This device could also be utilized to validate gating by making a single recording and composite of all detections between beam holds. Lower dose rate measurements would also require an additional measurement as there is no way to combine that with any other measurement with this or any type of detector.

Output measurements for this device show good agreement and sensitivity to small changes using a calibration curve compared to the TPS and the measured DailyQA3. The DaiilyQA3 device also correlated well with monthly or annual ionization chamber measurement on average of 0.02% +/0.14%. A single calibration curve can be used for all energies since the response of the plastic scintillator is energy independent as shown above and discussed in the literature.^7^, ^23^, ^26^ 10xFFF consistently had a higher measurement with multiple setups, but the 15x measurements agreed with less than 1% difference. The noted deviations in the 10xFFF measurements could be attributed to the higher dose rate of 2400MU/min rather than 600MU/min of the pulsed 10xFFF beam in relation to the frame rate of the camera. For dose measurements, it is very important to ensure the same camera settings are used in each measurement, particularly the gain and exposure time. Adjusting the gain changes the amplification of the signal, which results in a different correlation between signal to dose measured. For relative measurements such as PDD or profiles, this change is not as impactful because the amplification of the signal is the same across the entire camera. A different exposure time would affect the amount of light collected. A separate calibration curve would need to be created. The length of the exposure time could affect the accuracy as well, since the Linac delivers pulses at 360Hz and if the camera settings are not optimal, random pulses could be missed. Increasing the exposure time to better match the frame rate would minimize the dead time for the camera and ensure a more reproducible measurement. For constant dose rate applications, such as output measurements, we saw this effect to be minimal, but for dynamic measurements minimizing the deadtime would be preferrable.

For profiles the deviations seen in the measured penumbra region for square fields are a result of both the field of view and the refraction of the distal portion of the beam being different than the proximal portion of the beam. Since the scintillation light from these regions travel different distances within the scintillator, and at different angles, the end effect is similar to that of a partial averaging effect for a detector scanning the penumbra. Figure 2a shows an example of how the light is summed in a pixel in the camera along the penumbra for part of the beam. This effect is more notably observed for larger field sizes than smaller ones due to the larger difference in distance traveled within the scintillator. The variation in the field of view for the depth of the camera is also greater. Without any depth information, this detector is currently limited in how refraction can be corrected for. The sum of light collected in a pixel has a cone beam geometry and we can only apply one refraction correction for each pixel. The comparison for QA measurements is to a baseline measurement, therefore this issue would not have an effect in a monthly QA program. The mismatch in penumbra is consistent from setup to setup as displayed in Figure 8. To verify the detector accuracy a narrow field is measured to minimize this refraction error. The rectangular field measurements showed better agreement in the penumbra region as observed in Figures 6 and 7. This study measured both rectangular fields and square fields to validate the detector as well as confirm its utilization for common quality assurance fields respectively.

Other commercial detectors have been utilized for efficient monthly QA such as the IC Profiler and the EPID detector. Taneja et al explored the IC Profiler use for output measurements and beam consistency through flatness and symmetry measurements as did Skinner et al.^5^, ^6^ Their results of the output consistency to be 0.16 ± 0.61% and 0.3% ± 0.3% respectively are consistent with the results presented here. Their flatness and symmetry results of 0.04% ± 0.1725% and 0.5% ± 0.1% respectively compare well to with our point‐by‐point comparison of mean difference of 0.05% ± 0.39%. Finally, the beam energy results of ‐0.02% ± 0.3% compares to the measurements in this study of 0.012%. The EPID detector has generally only been used for monthly QA to evaluate mechanicals, MLC positioning and light vs rad as described by Eckhause et al.^27^ EPID use for dosimetric parameter measurements has been limited to daily QA, but has shown a consistency of 0.33% ± 0.26% for output and 0.5% ‐1.2% for flatness and symmetry.^28^

Future work on this detector is planned to explore reconstruction of dose in 3 dimensions (3D), in which the LINAC's electronic portal imaging device (EPID) will provide depth information. The orthogonal projection to the scintillator image allows for reconstructing dose in 3D. The refraction correction method will be simplified and more accurate since additional depth information will allow the refraction to be corrected voxel by voxel. The integration of the EPID would also reduce the measurements needed to complete monthly QA, as the additional image with the couch rotation, as described above, can be provided with the data from the EPID.

## 5 CONCLUSIONS

The results of this study show that a plastic scintillator detector can provide an efficient and effective technique to measure all dosimetric parameters relevant to monthly QA as described in AAPM TG reports and MPPG guidelines.^1^, ^2^, ^3^, ^4^ The accuracy of the measurements studied are shown to be reproducible and agree within 2%/2mm 1D gamma calculations of the TPS generated baseline for PDD and crossline profiles. Output measurements show less than 1.3% difference compared to the TPS. All measurements were reproducible on average to less than 0.2% difference with 0.3% standard deviation.

## AUTHOR CONTRIBUTION

All authors discussed the results and contributed to the final manuscript.

## CONFLICT OF INTEREST STATEMENT

The authors have nothing to report.
